# Nobiletin, a polymethoxylated flavonoid, regulates cell survival via the nuclear receptor RORα in cardiomyocytes

**DOI:** 10.20517/jca.2023.46

**Published:** 2023-12-31

**Authors:** Yuka Shiheido-Watanabe, Junichi Sadoshima

**Affiliations:** Rutgers New Jersey Medical School, Department of Cell Biology and Molecular Medicine Rutgers, New Jersey Medical School, Newark, NJ 07103, USA.

Cardiomyocyte homeostasis is maintained via the proper removal of unwanted proteins and damaged mitochondria by autophagy and mitophagy, respectively, thereby protecting the heart against various stresses. When autophagy/mitophagy is reduced, however, both protein quality and mitochondrial function deteriorate, leading to heart failure. Thus, developing an intervention that maintains autophagy and mitophagy function appears to be a promising strategy for heart failure therapeutics.

Nuclear receptors have been recognized as central transcriptional regulators of cardiomyocyte mitochondrial structure and function^[1]^. Among these receptors, retinoic acid-related orphan receptors (RORs) have emerged as noteworthy transcription factors. Three subtypes of RORs are known to exist: RORα, RORβ, and RORγ. In particular, RORα is widely expressed in various tissues, including the heart^[2]^. RORα plays an important role in the regulation of circadian rhythm and metabolism, as well as T cell development. RORα plays a protective role against the progression of non-alcoholic fatty liver disease through modulation of mitochondrial dynamics^[3]^. RORα also protects neurons against oxidative stress-induced apoptosis^[4]^. In the heart, RORα deficiency exacerbates high-fat diet-induced myocardial dysfunction by impairing mitochondrial biogenesis and function^[2]^. Thus, activation of RORα has been considered a potential therapeutic intervention in human diseases^[1]^.

In the recent issue of *The Journal of Cardiovascular Aging*, Kirshenbaum *et al.* showed that Nobiletin, a polymethoxylated flavonoid, promotes cell survival in cardiomyocytes by stimulating RORα [Figure 1]^[5]^. Nobiletin is known to exert a wide variety of pharmacological effects, including protection against inflammation, oxidative stress, and tumor growth. Kirshenbaum *et al.* have shown that Nobiletin inhibits hypoxia-induced increases in mRNA levels of p16, a marker of senescence, and reactive oxygen species (ROS) production, thereby reducing cell death in cardiomyocytes^[5]^. Importantly, Nobiletin failed to rescue cardiomyocyte death in the absence of RORα, suggesting that RORα plays an important role in mediating the protective action of Nobiletin. Interestingly, Nobiletin also failed to suppress cell death in the absence of ATG7, an essential mediator of autophagy. Together with a previous study showing that Nobiletin stimulates lysosomal acidification in cardiomyocytes^[6]^, the study by Kirshenbaum *et al.* suggests that Nobiletin and RORα exert their cardioprotection at least in part through activation of autophagy/mitophagy^[5]^.

The study by Kirshenbaum *et al.* demonstrates the possibility of using Nobiletin or RORα agonists to stimulate autophagy/mitophagy. However, several issues require clarification^[5]^. First, the mechanism through which Nobiletin protects the heart requires further investigation. Since the cardioprotective effect of Nobiletin is attenuated but not eliminated by downregulation of RORα, Nobiletin presumably promotes additional cardioprotective mechanisms besides stimulation of RORα. The authors showed that Nobiletin alleviates oxidative stress and improves mitochondrial function. Although these beneficial effects could be the consequence of autophagy and mitophagy activation by Nobiletin, it is equally possible that they are the cause of autophagy/mitophagy activation and that autophagy/mitophagy activation is a secondary effect. Second, since either downregulation or insufficient activation of autophagy/mitophagy is a common driver of aging and heart failure, it would be interesting to further investigate whether Nobiletin and stimulation of RORα can enhance or reactivate autophagy and mitophagy in other cardiac conditions, including natural aging *in vivo*. Third, although the authors proposed that RORα controls autophagic genes through direct binding to their promoters, a previous study in cardiomyocytes showed that RORα activates mitophagy through upregulation of caveolin-3^[7]^, which is not an autophagy gene. Thus, further characterization of the mechanisms through which RORα stimulates autophagy/mitophagy, including identification of direct target genes through ChIP-sequencing analyses, is required. Finally, it will be important to evaluate how the endogenous activity of RORα in cardiomyocytes is altered during pathological conditions and aging and how the level of autophagic flux is modulated by interventions that modulate RORα. In particular, the assessment of autophagic flux should be conducted using multiple methods.

In conclusion, the recent study by Kirshenbaum *et al*. showed that Nobiletin exerts a protective effect against stress through the activation of RORα in cardiomyocytes^[5]^. Identifying direct transcriptional targets of RORα may lead to the discovery of novel stimulators of autophagy and mitophagy that can be used for the treatment of a variety of cardiovascular diseases. If RORα and its downstream mediators stimulate autophagy/mitophagy through a unique molecular mechanism not utilized by other known stimulators, using them in conjunction with other stimulators of autophagy/mitophagy would be expected to have an additive effect. Finally, since RORα is generally protective not only in the heart but also in other organs, small molecule agonists that selectively stimulate RORα should have wide therapeutic applications not limited to cardiovascular disease.

## Figures and Tables

**Figure 1.**
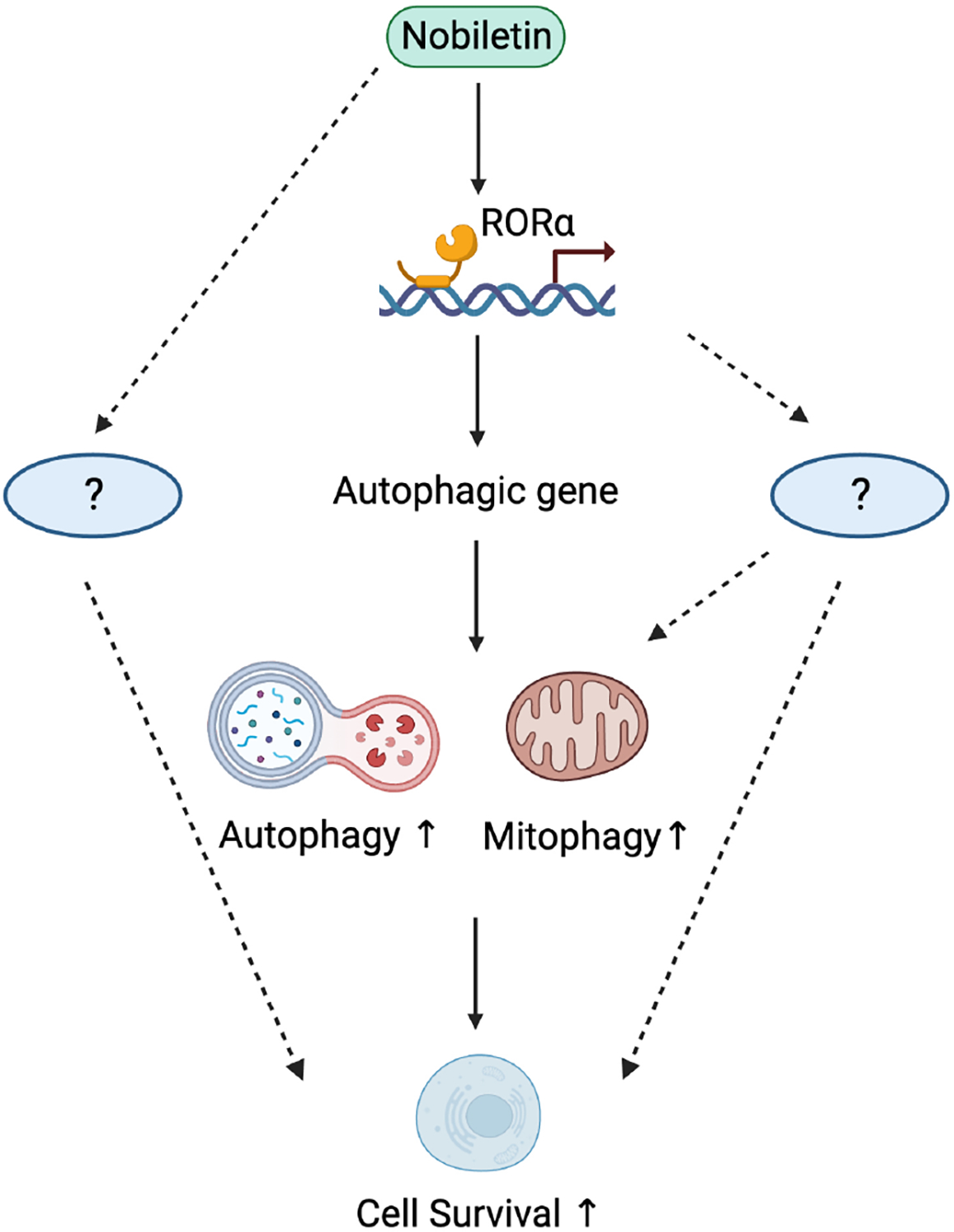
A model of the protective role of Nobiletin via RORα activation. Nobiletin activates RORα, which activates autophagy/mitophagy, leading to cell protection. It should be noted that Nobiletin may induce cell survival independently of RORα, whereas RORα’s stimulatory effect of upon may be secondary to unknown mechanisms.

## Data Availability

Not applicable.
